# Severe pulmonary radiological manifestations are associated with a distinct biochemical profile in blood of tuberculosis patients with dysglycemia

**DOI:** 10.1186/s12879-020-4843-0

**Published:** 2020-02-14

**Authors:** Nadia Nilda Barreda, Maria B. Arriaga, Juan Gonzalo Aliaga, Kattya Lopez, Oswaldo Martin Sanabria, Thomas A. Carmo, José F. Fróes Neto, Leonid Lecca, Bruno B. Andrade, Roger I. Calderon

**Affiliations:** 1 Socios En Salud Sucursal Peru, 15001 Lima, Peru; 2 Universidad Nacional Mayor de San Marcos, Lima, 15081 Peru; 3 Faculdade de Medicina, Universidade Federal da Bahia, Salvador, Bahia 40110-100 Brazil; 4 Instituto Brasileiro para Investigação da Tuberculose, Fundação José Silveira, Salvador, Bahia 40210-320 Brazil; 5 Instituto Gonçalo Moniz, Fundação Oswaldo Cruz, Salvador, Bahia 40269-710 Brazil; 6 Multinational Organization Network Sponsoring Translational and Epidemiological Research (MONSTER) Initiative, Fundação José Silveira, Salvador, Bahia 40210-320 Brazil; 7 Division of Rheumatology, Inflammation, and Immunity, Brigham and Women’s Hospital, Harvard Medical School, Boston, MA USA; 8 Universidade Salvador (UNIFACS), Salvador, Bahia 41720-200 Brazil; 9 Escola Bahiana de Medicina e Saúde Pública (EBMSP), Salvador, Bahia 40290-000 Brazil; 10 Curso de Medicina, Faculdade de Tecnologia e Ciências (FTC), 41, Salvador, Bahia 741-590 Brazil; 11 Faculdade de Medicina, Universidade Federal do Rio de Janeiro, Rio de Janeiro, 21941-590 Brazil

**Keywords:** Chest x-ray, Hyperglycemia, Diabetes mellitus, Prediabetes, Pulmonary tuberculosis

## Abstract

**Background:**

Diabetes mellitus (DM) is thought to affect tuberculosis (TB) clinical presentation and treatment response. Whether DM impacts radiological manifestations of pulmonary TB is still not clear. This study investigated the impact of glycemic status on radiological manifestations of pulmonary TB cases and its relationship with concentration of biochemical parameters in peripheral blood.

**Methods:**

A retrospective cross-sectional study used data from 132 microbiologically confirmed pulmonary TB patients from Lima, Peru, evaluated in a previous investigation performed between February and December 2017. Chest radiographs were analyzed by a radiologist and a pulmonologist. Radiographic lesions were identified as cavities, alveolar infiltrates and fibrous tracts. Hyperglycemia in TB patients was identified by use of fasting plasma glucose, HbA1c and oral glucose tolerance test. Clinical, biochemical and hematological parameters were also analyzed.

**Results:**

TB patients with hyperglycemia presented more frequently with cavities, alveolar infiltrates and fibrous tracts than those with normoglycemia. Hierarchical clustering analysis indicated that patients with more diverse and higher number of lung lesions exhibited a distinct laboratorial profile characterized by heightened white blood cell counts and circulating levels of total cholesterol, triglycerides and transaminases and simultaneously low levels of albumin and hemoglobin. Multivariable regression analyses adjusted for age, sex, prior TB, hemoglobin levels and acid-fast bacilli ≥2+ in sputum smears, demonstrated that presence of prediabetes or diabetes in TB patients was associated with increased odds of having 3 pulmonary lesion types (*p* = 0.003 and *p* < 0.01 respectively) or ≥ 4 lesions (*p* = 0.001 and *p* = 0.01 respectively).

**Conclusion:**

Hyperglycemia (both DM and prediabetes) significantly affected the presentation of radiographic manifestations and the number of lesions in pulmonary TB patients as well as the biochemical profile in peripheral blood.

## Background

The association between diabetes mellitus (DM) and tuberculosis (TB) has been re-called to attention in the 1980s, when the global prevalence of DM in adults increased by 20% in less than 30 years [1]. In recent years, hyperglycemia, which includes diabetes mellitus (DM) and prediabetes (PDM), has been reported to be very frequent in low and middle-income countries [2, 3] many of which are also endemic for TB [4, 5]. In Peru; this comorbidity has been recognized as an important issue since it challenges the success of TB control programs [6]. The Peruvian Ministry of Health has reported that the prevalence of DM in TB patients recently increased from 4 to 6.2% [7]. Conversely, in a recent study in Lima [8], we reported a much higher prevalence of DM (14%) and of PDM (31%) in TB patients. Both local and international agencies recommend both continuous screening for dysglycemia and tight glycemic control in people with active TB [9]. However, several factors limit the ability of individuals to properly monitor glycemic status/control [10, 11].

The mechanisms underlying the clinical outcomes of patients with TB-DM comorbidity are poorly understood. Poor glycemic control seems to exacerbate the clinical presentation of TB [12–14], increasing the frequency symptoms [11] and the radiographic manifestations of pulmonary TB [15]. Other studies, however, have failed to demonstrate that DM impacts radiographic signs of TB [16]. The radiology of thorax remains essential in the diagnosis of pulmonary TB. The clinical and radiological manifestations of pulmonary TB depend on the immune status of the infected host. Individuals with impaired immune responses, as observed in advanced HIV coinfection, commonly present with aberrant clinical and radiographic disease manifestations [17]. In different studies, DM has also been associated with a higher frequency of atypical radiological findings and cavitary tuberculosis [15], but there is still no consensus on whether TB-DM patients have worse radiographic disease. While some investigations have failed to demonstrate differences in frequency of lower lung lesions in TB-DM patients compared to those with normoglycemia [15], other studies have actually reported the opposite [18], suggesting that DM actually results in higher occurrence of typical cavitary lesions in TB patients [15, 18]. To better elucidate this question, we performed a study in which systematic radiographic evaluation was performed by both a radiologist and a pneumologist to describe the lung lesions in more details. We hypothesized that the radiographic manifestation of pulmonary TB is likely to be affected by dysglycemia (DM or PDM) in terms of types and number of lesions in patients from Peru.

## Methods

### Study design and settings

**Fig. 1:**
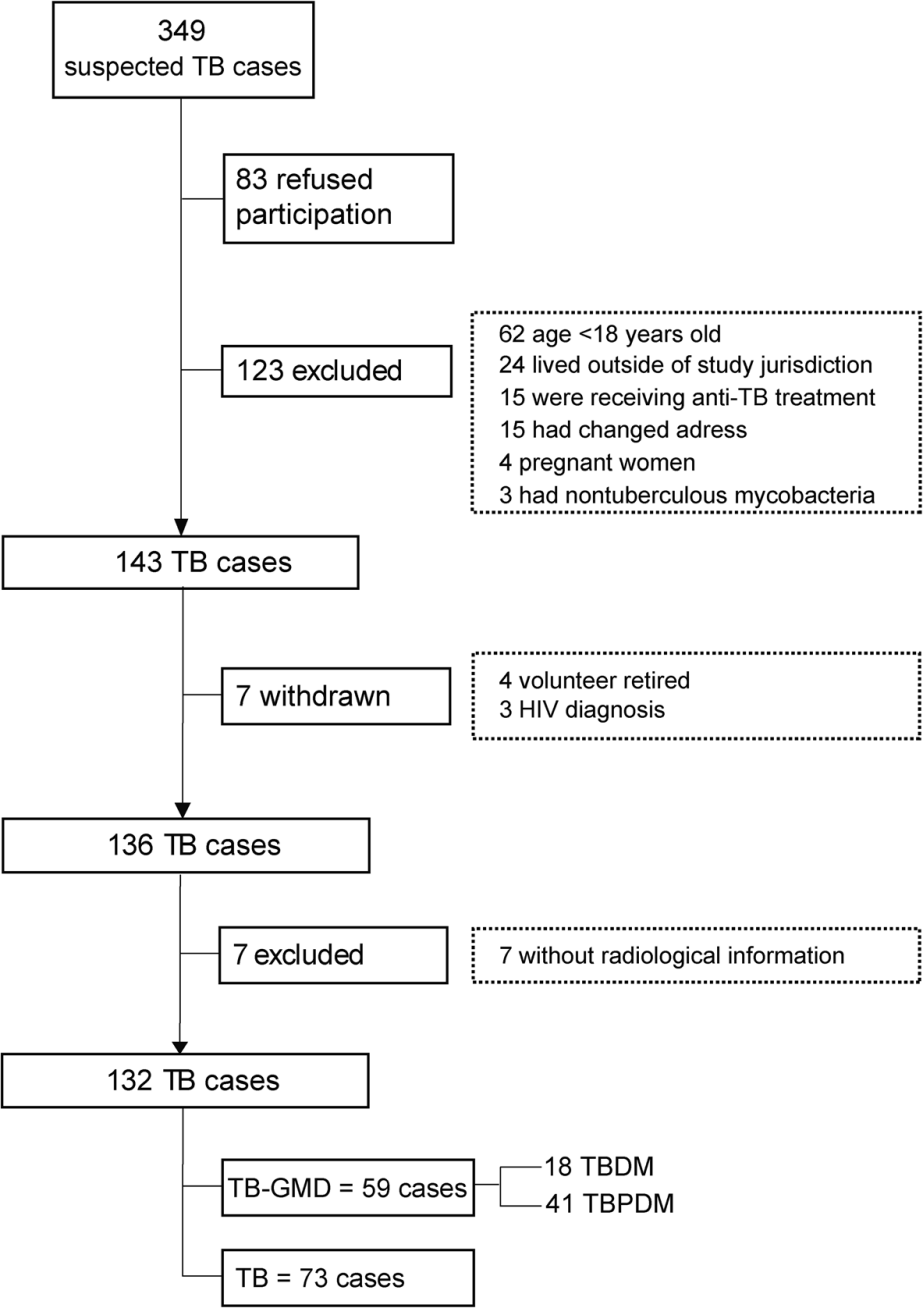
Study flowchart**.** TB: tuberculosis, GMD: glucose metabolism disorders, DM: diabetes mellitus, PDM: prediabetes

We performed a retrospective cross-sectional analysis of data collected originally from a larger prospective cohort study conducted between February and November 2017, which objective was to determine the prevalence of hyperglycemia in individuals with microbiologically confirmed pulmonary TB and their contacts [8]. For the present study, inclusion criteria were age ≥ 18 years of age, diagnosis performed by the National TB Program, patients who were not receiving anti-TB treatment or had started in no more than 5 days prior, and who had recorded chest X-rays analysis. The reason for excluding patients taking anti-TB treatment for more than 5 days was to minimize the exposure to the drugs, which has been shown to impact the glycemic status of patients after at least 2 weeks [19]. The study inclusion details of TB patients are outlined in Fig. 1. More information on patient evaluation, interviews and procedures have been published previously [8].

### Laboratory and field procedures

All laboratory procedures were described in a previous publication [8]. All these tests were performed at the Socios En Salud (SES) Laboratory, located in Lima and graded following the standard recommendations [20–22]. Briefly, in all sputum samples, a direct examination (smear) was performed using the Ziehl-Neelsen staining and the semi-quantitative results were recorded. In parallel, a portion of the sputum samples was decontaminated with NAL-NaOH and then seeded in Lowenstein-Jensen medium or cultured using the BD MGIT 960 System (liquid culture). Confirmation of cultures which were positive for Mtb and drug sensitivity testing was then performed for the first line drugs rifampicin, isoniazid, ethambutol, streptomycin and pyrazinamide.

A panel composed by a pulmonologist and a radiologist analyzed and documented the readings from chest radiographs taken at the diagnosis. Cavities, infiltrates and fibrous tract were documented for the medical assessment in all study participants, based on Peruvian TB program guidelines [9]. Moreover, data on white blood cells count (WBC) as well as serum levels of hemoglobin (Hb), cholesterol (Chol), triglycerides (TG); alanine aminotransferase (ALT), aspartate aminotransferase (AST) and albumin were retrieved, reviewed and analyzed from the electronic medical records.

To determine the association of radiological manifestations with hyperglycemia, diagnosis of DM or prediabetes (PDM) was performed in agreement with American Diabetes Association (ADA) guidelines [4], and was based on fasting plasma glucose (FPG), glycated hemoglobin (HbA1c) and oral glucose tolerance test (OGTT) as previously described [8]. The measurement of HbA1c in whole blood specimens was performed by use of TRI-stat™ platform (Trinity Biotech, Ireland) and FPG or glucose level on OGTT were performed following standard methods. Of note, OGTT was not performed when the patient had already diagnosis of DM due to safety issues raised by the Institutional Review Board (IRB) which handled the Ethical aspects of the study protocol. Anemia was defined following WHO criteria as hemoglobin (Hb) level below 12.5 g/dL for female and 13.5 g/dL for male. The SES lab conducts annual external quality assurance through competition panels of the College of American Pathologists (Northfield, Illinois) and other agencies.

### Clinical data

Information on socio-demographic and clinical evaluation was retrieved from the medical records. Hypertension was defined according to WHO criteria with measurements taken on two different days, demonstrating systolic blood pressure readings ≥140 mmHg and/or the diastolic blood pressure readings ≥90 mmHg on both days. Anthropometric assessment was also performed including weight, height and abdominal circumference measurement. Socios En Salud Informatic System (SEIS) software (Lima, Peru) was used to management of the demographic and clinical information.

### Data analysis

Characteristics of study participants were presented as median and interquartile range (IQR) values for continuous variables or frequency for categorical variables. Continuous variables were compared using the Mann-Whitney *U* test (between two groups) or the Kruskal-Wallis test with Dunn’s multiple comparisons (between > 2 groups). Categorical variables were compared using the Fisher’s exact test (2 × 2 comparisons) or Pearson’s chi square test. We also performed additional analyses employing dimension reduction approaches to define an overall biochemical profile associated with distinct radiographic lung lesions. Therefore, biochemical data were z-score normalized across the entire cohort and analyzed by hierarchical clustering (Ward’s method, with 100X bootstrap). In this approach, dendrograms represented Euclidean distance, which infers similarity between the individuals and groups of study participants [23]. A multivariable regression model using variables with univariate *p*-value ≤0.2 was performed to assess the odds ratios (OR) and 95% confidence intervals (CIs) of the associations with 3 types lesion and the number of lung lesions ≥4 (defined here as outcomes). All analyses were pre-specified. Two-sided *p* value < 0.05 were considered statistically significant. Since our population composed by TB patients affected by hyperglycemia (59) and not affected by hyperglycemia (73), the study power was 95% (alpha risk of 0.05 in a bilateral contrast) to detect as statistically significant differences in worse radiological manifestations at ratios of 0.586 and 0.260 respectively. Statistical analyses were performed using SPSS 24.0 (IBM statistics), Graphpad Prism 7.0 (GraphPad Software, San Diego, CA) and JMP 13.0 (SAS, Cary, NC, USA).

## Results

In this study, from 349 microbiologically confirmed TB cases initially screened at the primary health care centers (Fig. 1), 206 individuals were excluded for a number of reasons listed in Fig. 1, and 143 patients with active TB were further examined. At this stage, additional 14 persons were excluded due to HIV diagnosis (*n* = 7) and for non-existence of radiography records (*n* = 7), resulting in a population of 132 patients who were included in this cross-sectional study. Of note, 59 (44.7%) patients were diagnosed with hyperglycemia, and from those, 41 (31.1%) had PDM and 18 (13.6%) had DM.

Individuals with DM were on average older than those with PDM or normoglycemia (median age: 46.1 yrs. IQR:36.6–58.3 vs. 40 yrs., IQR: 26.7–54.0 and 25.8 yrs. IQR: 21.0–30.9, respectively, *p* < 0.01) (Table 1). Lower level of education (elementary and secondary school years) was associated with presence of hyperglycemia (DM or PDM) (*p* < 0.01) (Table 1). The frequencies of both history of TB (66.7%) and hypertension (16.7%) were more frequent in DM patients than in PDM and normoglycemic individuals (*p* < 0.01). The DM, PDM and normoglycemic groups were similar with regard to a number of other characteristics including sex, BCG vaccination, history of asthma and renal disease, as well as life-style habits such as use of alcohol, illicit drugs or smoking. Furthermore, frequency of increased acid-fast bacilli grades in sputum smears was higher in the group of DM cases compared to the other groups (*p* = 0.01) (Table 1).

**Table 1:** Characteristics of pulmonary TB cases stratified according to DM status in Lima, Peru, 2017

Characteristics	DM *n* = 18	PDM *n* = 41	Normoglycemia *n* = 73	*p*-value
Age (years)-median (IQR)	46.15 (36.64–58.28)	40 (26.67–53.89)	25.83 (21.05–30.92)	< 0.01
Sex				0.42
Male	8 (44.4)	28 (68.3)	45 (61.6)	
Female	10 (55.6)	13 (31.7)	28 (38.4)	
Education				< 0.01
Elementary and secondary school	17 (94.4)	36 (87.8)	45 (61.6)	
Higher education	1 (5.6)	5 (12.2)	28 (38.4)	
Prior TB	12 (66.7)	2 (4.9)	2 (2.7)	< 0.01
BCG vaccination	16 (88.9)	37 (92.5)	69 (94.5)	0.40
Smoking	4 (22.2)	9 (22.5)	15 (20.5)	0.82
Smoker at home	2 (11.1)	4 (10)	5 (6.8)	0.48
Cannabis use	1 (5.6)	6 (15)	13 (17.8)	0.23
Illicit drug use	1 (5.6)	7 (17.5)	8 (11)	0.92
Alcohol use	3 (16.7)	27 (67.5)	37 (50.7)	0.16
Hypertension	3 (16.7)	4 (10)	0 (0)	< 0.01
Asthma	0 (0)	3 (7.5)	4 (5.5)	0.57
Renal disease	1 (5.6)	0 (0)	1 (1.4)	0.41
Slow scarring	3 (16.7)	9 (22.5)	7 (9.6)	0.17
Metformin use	6 (33.3)	1 (2.7)	0 (0)	< 0.01
BMI (kg/m2)-median (IQR)	22.43 (21.41–26.36)	23.39 (21.53–25.04)	22.31 (20.25–25.39)	0.74
Waist circumference (cm) -median (IQR)	84 (80–89)	84 (77–90)	80 (74–86)	0.04
Hemoglobin (g/dL) -median (IQR)	10.55 (9.9–11.2)	11.8 (10.35–13.1)	12.6 (11.25–13.4)	< 0.01
FPG (mg /dL) -median (IQR)	259.55 (155.3–311.6)	100.4 (95.3–103.7)	89.9 (85.7–94.5)	< 0.01
HbA1c (%)- median (IQR)	11 (9.1–13.5)	5.3 (5–5.65)	5 (4.7–5.2)	< 0.01
OGTT (mg/dL) -median (IQR)	119.5 (119.5–119.5)	128.45 (110.3–157.05)	105 (83.3–122)	< 0.01
AFB smear				0.01
Negative	5 (27.8)	14 (34.1)	38 (52.8)	
1+	3 (16.7)	9 (22)	15 (20.8)	
2+	3 (16.7)	5 (12.2)	6 (8.3)	
3+	6 (33.3)	11 (26.8)	11 (15.3)	
Scanty	1 (5.6)	2 (4.9)	2 (2.8)	
L-J culture				1.00
Negative	4 (22.2)	8 (20.5)	24 (34.3)	
1+	11 (61.1)	18 (46.2)	32 (45.7)	
2+	2 (11.1)	4 (10.3)	3 (4.3)	
3+	0 (0)	4 (10.3)	3 (4.3)	
colonies	1 (5.6)	5 (12.8)	8 (11.4)	
BD MGIT™ 960 System				0.49
Positive	8 (80)	22 (81.5)	34 (87.2)	
Negative	2 (20)	5 (18.5)	5 (12.8)	
MDR	2 (18.2)	3 (12)	4 (10)	0.49
Isoniazid -resistant	2 (18.2)	5 (20)	7 (17.5)	0.89
Rifampicin -resistant	2 (18.2)	4 (16)	5 (12.5)	0.59
Cough for more than 4 weeks	17 (94.4)	38 (92.7)	65 (89)	0.40
Hemoptysis	8 (44.4)	13 (31.7)	34 (46.6)	0.45
Fever	6 (33.3)	20 (48.8)	35 (47.9)	0.39
Dyspnea	12 (66.7)	25 (61)	49 (67.1)	0.77
Night sweats	12 (66.7)	22 (53.7)	41 (56.2)	0.58
No appetite	12 (66.7)	28 (68.3)	40 (54.8)	0.19
Lost weight	15 (83.3)	35 (85.4)	47 (64.4)	< 0.01
Fatigue	15 (83.3)	33 (80.5)	55 (75.3)	0.40
Polyuria	8 (44.4)	17 (41.5)	26 (35.6)	0.42
Polydipsia	8 (44.4)	21 (51.2)	34 (46.6)	0.95
Type lesions				< 0.01
1	0 (0)	9 (22)	30 (41.1)	
2	4 (22.2)	12 (29.3)	24 (32.9)	
3	14 (77.8)	20 (48.8)	19 (26)	
Number of lesions - mean ± SD	6.1 ± 2.2	4.1 ± 1.8	2 ± 8	< 0.01
Number of lesions				< 0.01
≥ 4 lesions	17 (94.4)	27 (65.9)	24 (32.9)	
< 4 lesions	1 (5.6)	14 (34.1)	49 (67.1)	

Data represent no. (%); *IQR* Interquartile range. Except Number of lesions represented in mean ± SD (standard deviation). *BCG* Bacillus Calmette–Guérin, *BMI* Body Mass Index, *Hb* Hemoglobin, *FPG* Fasting Plasma Glucose, *HbA1c* Glycated Hemoglobin, *OGTT* Oral Glucose Tolerance Test, *AFB* Acid-Fast Bacilli, *L-J* Löwenstein-Jensen, *MDR* Multi Drug Resistant. Hypertension, asthma, renal disease and anemia as defined by the World Health Organization as described in Methods. Prior TB: diagnosis of active tuberculosis before of this

**Fig. 2:**
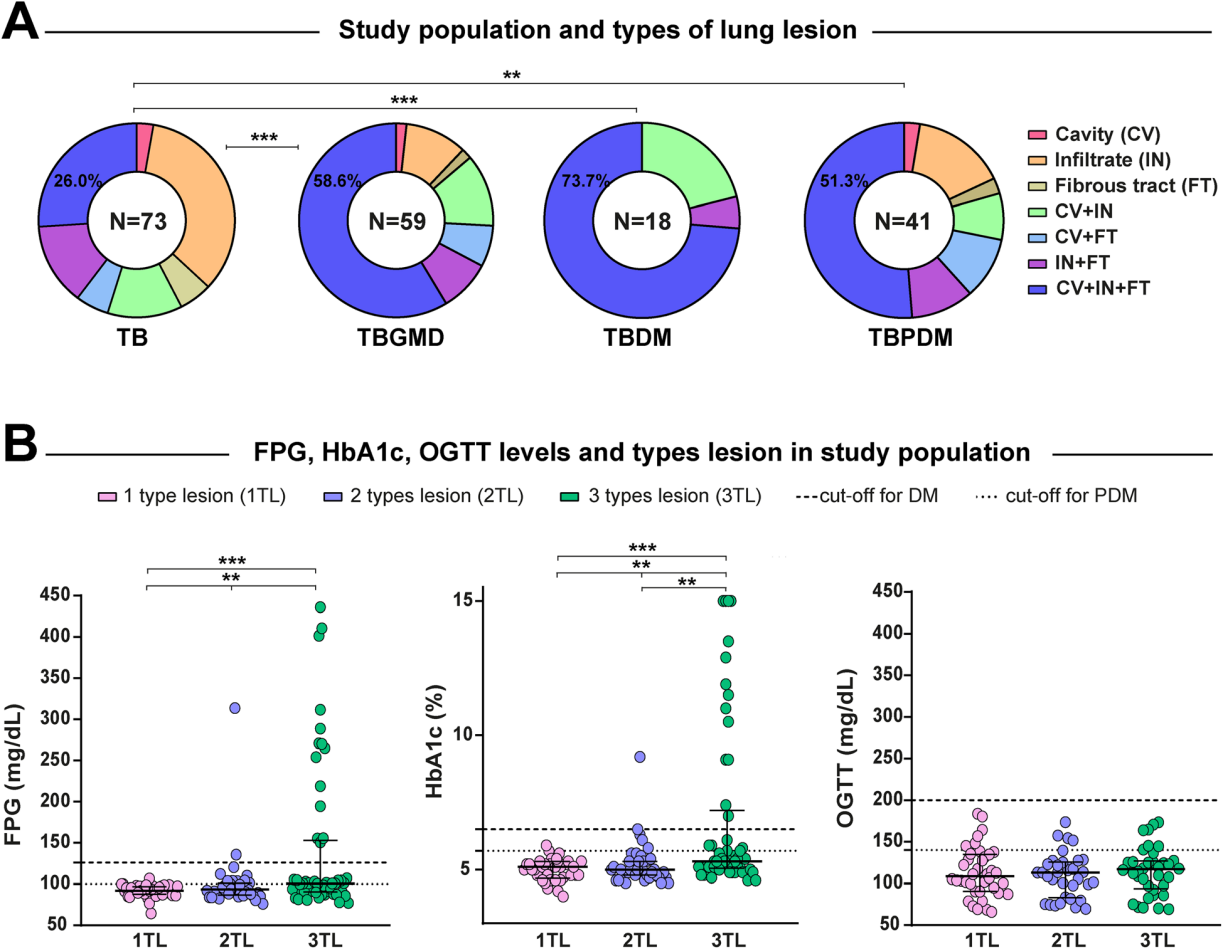
Distribution of lung lesions according to levels of FPG, HbA1c and OGTT in TB patients. **a** Distribution of lung lesions according to the glycemic status of TB patients. **b** Scatter plots depicting the distribution of FPG, HbA1c and OGTT values in TB patients with 1, 2 or 3 types of lesions. Lines represent median and interquartile range values. The differences in median values (and IQR) of glucose, HbA1c and OGGT between groups were compared using the Kruskal-Wallis test with Dunn’s multiple comparisons post-test. TB: tuberculosis, GMD: glucose metabolism disorders, DM: diabetes mellitus PDM: prediabetes, FPG: Fasting plasma glucose, HbA1c: glycated hemoglobin, OGTT: oral glucose tolerance test. One type lesion: cavity (CV) or infiltrate (IN) or fibrous tract (FT); 2 lesion types: CV + IN or CV + FT or IN + FT; 3 lesion types: CV + IN + FT. Number of lung lesions ≥4: considering the total number of cavities + total number of infiltrates + total number of fibrous tracts. Only comparisons with significant *p*-values are displayed (*p < 0.05, **p < 0.01, ***p < 0.001).

The statistical analysis of radiographic examination revealed that the absolute number of lung lesions as well as the frequency of different lesion types were both higher in the DM group compared to the groups of PDM and normoglycemic (*p* < 0.01) (Table 1). No differences were found between the characteristics of pulmonary TB cases stratified according to the presentation of the types of lung lesions or number of lesions, with the exception of previously treated TB (Tables 2 and 3, respectively). Simultaneous presence of the 3 different types of lung lesions (cavities, infiltrates and fibrous tracts), was significantly higher among hyperglycemic vs. normoglycemic TB patients (59 and 26% respectively, *p* < 0.001; Fig. 2a). Such frequency of the three lesion types was not different between DM (73.7%) and PDM (51.3%) TB patients (Fig. 2a).

**Table 2:** Characteristics of TB patients stratified according to types of lung lesions in Lima, Peru, 2017

Characteristics	1 Type lesion *n* = 39	2 Type lesions *n* = 40	3 Type lesions *n* = 53	*p*-value
Age (years)-median (IQR)	27.93 (22.75–31.92)	28.10 (22.04–49.06)	37.92 (24.58–47.64)	0.07
Sex				0.94
Male	23 (59.0)	25 (62.5)	33 (62.3)	
Female	16 (41.0)	15 (37.5)	20 (37.7)	
Education				0.12
Elementary and secondary school	25 (64.1)	29 (72.5)	44 (83.0)	
Higher education	14 (35.9)	11 (27.5)	9 (17.0)	
Prior TB	1 (2.6)	3 (7.5)	12 (22.6)	< 0.01
BCG vaccination	35 (89.7)	39 (97.5)	48 (92.3)	0.38
Smoking	7 (17.9)	13 (32.5)	8 (15.4)	0.12
Smoker at home	5 (12.8)	1 (2.5)	5 (9.6)	0.24
Cannabis use	6 (15.4)	8 (20.0)	6 (11.5)	0.54
Illicit drug use	3 (7.7)	7 (17.5)	6 (11.5)	0.41
Alcohol use	21 (53.8)	23 (57.5)	23 (44.2)	0.42
Hypertension	1 (2.6)	3 (7.5)	3 (5.8)	0.61
Asthma	4 (10.3)	1 (2.5)	2 (3.8)	0.26
Renal disease	0 (0.0)	1 (2.5)	1 (1.9)	0.64
Slow scarring	6 (15.4)	5 (12.5)	8 (15.4)	0.91
Metformin use	0 (0.0)	2 (5.3)	5 (9.8)	0.13
BMI (kg/m2)-median (IQR)	23.53 (21.22–25.08)	22.28 (20.08–25.39)	22.66 (20.19–25.68)	0.28
Waist circumference (cm) -median (IQR)	84 (76–90)	81 (75–86)	82 (77–89)	0.34
Hemoglobin (g/dL) -median (IQR)	12.60 (11.50–13.30)	12.15 (10.85–13.53)	11.25 (10.53–12.65)	0.03
FPG (mg /dL) -median (IQR)	91.5 (7.3)	100.4 (36.3)	140.9 (91.7)	< 0.01
HbA1c (%)- median (IQR)	4.9 (0.41)	5.2 (0.8)	6.9 (3.2)	< 0.01
OGTT (mg/dL) -median (IQR)	113.4 (29.9)	109.8 (26.7)	115.0 (28.6)	0.76
AFB smear				0.04
Negative	23 (59.0)	17 (43.6)	17 (32.1)	
1+	8 (20.5)	7 (17.9)	12 (22.6)	
2+	1 (2.6)	6 (15.4)	7 (13.2)	
3+	6 (15.4)	7 (17.9)	15 (28.3)	
Scanty	1 (2.6)	2 (5.1)	2 (3.8)	
L-J culture				< 0.01
Negative	14 (36.8)	16 (43.2)	6 (11.5)	
1+	18 (47.4)	14 (37.8)	29 (55.8)	
2+	1 (2.6)	4 (10.8)	4 (7.7)	
3+	1 (2.6)	2 (5.4)	4 (7.7)	
Colonies	4 (10.5)	1 (2.7)	9 (17.3)	
BD MGIT™ 960 System				0.43
Positive	18 (81.8)	13 (65.0)	33 (97.1)	
Negative	4 (18.2)	7 (35.0)	1 (2.9)	
MDR	3 (14.3)	2 (12.5)	4 (10.3)	0.90
Isoniazid -resistant	4 (19.0)	2 (12.5)	8 (20.5)	0.78
Rifampicin -resistant	4 (19.0)	3 (18.8)	4 (10.3)	0.57
Cough for more than 4 weeks	33 (84.6)	37 (92.5)	50 (94.3)	0.26
Hemoptysis	15 (38.5)	17 (42.5)	23 (43.4)	0.89
Fever	21 (53.8)	18 (45.0)	22 (41.5)	0.50
Dyspnea	29 (74.4)	27 (67.5)	30 (56.6)	0.20
Night sweats	20 (51.3)	22 (55.0)	33 (62.3)	0.56
No appetite	23 (59.0)	26 (65.0)	31 (58.5)	0.79
Lost weight	28 (71.8)	29 (72.5)	40 (75.5)	0.91
Fatigue	30 (76.9)	29 (72.5)	44 (83.0)	0.47
Polyuria	15 (38.5)	13 (32.5)	23 (43.4)	0.57
Polydipsia	16 (41.0)	21 (52.5)	26 (49.1)	0.58

Data represent no. (%); *IQR* Interquartile range. *BCG* Bacillus Calmette–Guérin, *BMI* Body Mass Index, *Hb* Hemoglobin, *FPG* Fasting Plasma Glucose, *HbA1c* Glycated Hemoglobin, *OGTT* Oral Glucose Tolerance Test, *AFB* Acid-Fast Bacilli, *L-J* Löwenstein-Jensen, *MDR* Multi Drug Resistant. Hypertension, asthma, renal disease and anemia as defined by the World Health Organization as described in Methods. Prior TB: diagnosis of active tuberculosis before of this

**Table 3:** Characteristics of pulmonary TB stratified according to number of lung lesions in Lima, Peru, 2017

Characteristics	≥ 4 lesions *n* = 68	< 4 lesions *n* = 64	*p*-value
Age (years)-median (IQR)	34.89 (24.32–47.85)	27.93 (22.75–35.84)	0.03
Sex			1.00
Male	42 (60.9)	39 (60.0)	
Female	27 (39.1)	26 (40.0)	
Education			0.17
Elementary and secondary school	55 (79.7)	45 (69.2)	
Higher education	14 (20.3)	20 (30.8)	
Prior TB	14 (20.3)	2 (3.1)	< 0.01
BCG vaccination	64 (94.1)	60 (92.3)	0.74
Smoking	14 (20.6)	14 (21.5)	1.00
Smokers at home	5 (7.4)	6 (9.2)	0.76
Cannabis use	7 (10.3)	13 (20.0)	0.15
Illicit drug use	8 (11.8)	8 (12.3)	1.00
Alcohol use	33 (48.5)	36 (55.4)	0.49
Hypertension	6 (8.8)	1 (1.5)	0.12
Asthma	1 (1.5)	6 (9.2)	0.06
Renal disease	1 (1.5)	1 (1.5)	1.00
Slow healing	9 (13.2)	10 (15.4)	0.81
Metformin use	7 (10.8)	0 (0)	0.01
BMI (kg/m2)-median (IQR)	22.52 (20.28–25.22)	23.25 (20.68–25.39)	0.32
Waist circumference (cm) -median (IQR)	82 (77–88)	83 (75–88)	0.99
Hemoglobin (g/dL) -median (IQR)	11.5 (10.3–12.95)	12.4 (11.23–13.23)	0.03
FPG (mg /dL) -median (IQR)	100.7 (91.9–110.1)	90.5 (86.9–96.7)	< 0.01
HbA1c (%)- median (IQR)	5.3 (4.9–6)	5.1 (4.8–5.3)	< 0.01
OGTT (mg/dL) -median (IQR)	118 (97.1–136.6)	111 (90.3–127.5)	0.25
AFB smear			0.02
Negative	25 (36.8)	34 (52.3)	
1+	13 (19.1)	14 (21.5)	
2+	8 (11.8)	6 (9.2)	
3+	18 (26.5)	10 (15.4)	
Scanty	4 (5.9)	1 (1.5)	
L-J culture			0.06
Negative	14 (21.5)	23 (35.9)	
1+	30 (46.2)	32 (50.0)	
2+	7 (10.8)	2 (3.1)	
3+	5 (7.7)	2 (3.1)	
Colonies	9 (13.8)	5 (7.8)	
BD MGIT™ 960 System			0.55
Positive	36 (85.7)	28 (80.0)	
Negative	6 (14.3)	7 (20.0)	
MDR	6 (14.3)	3 (8.8)	0.72
Isoniazid-resistant	7 (16.7)	7 (20.6)	0.77
Rifampicin-resistant	7 (16.7)	4 (11.8)	0.75
Cough for more than 4 weeks	63 (91.3)	58 (89.2)	0.78
Hemoptysis	30 (43.5)	27 (41.5)	0.86
Fever	28 (40.6)	34 (52.3)	0.23
Dyspnea	39 (56.5)	48 (73.8)	0.05
Night sweats	39 (56.5)	37 (56.9)	1.00
No appetite	39 (56.5)	42 (64.6)	0.38
Lost weight	48 (69.6)	49 (75.4)	0.56
Fatigue	55 (79.7)	49 (75.4)	0.68
Polyuria	27 (39.1)	25 (38.5)	1.00
Polydipsia	31 (44.9)	32 (49.2)	0.73

Data represent no. (%); *IQR* Interquartile range. *BCG* Bacillus Calmette–Guérin, *BMI* Body Mass Index, *Hb* Hemoglobin, *FPG* Fasting Plasma Glucose, *HbA1c* Glycated Hemoglobin, *OGTT* Oral Glucose Tolerance Test, *AFB* Acid-Fast Bacilli, *L-J* Löwenstein-Jensen, *MDR* Multi Drug Resistant. Hypertension, asthma, renal disease and anemia as defined by the World Health Organization as described in Methods. Prior TB: diagnosis of active tuberculosis before of this

**Fig. 3:**
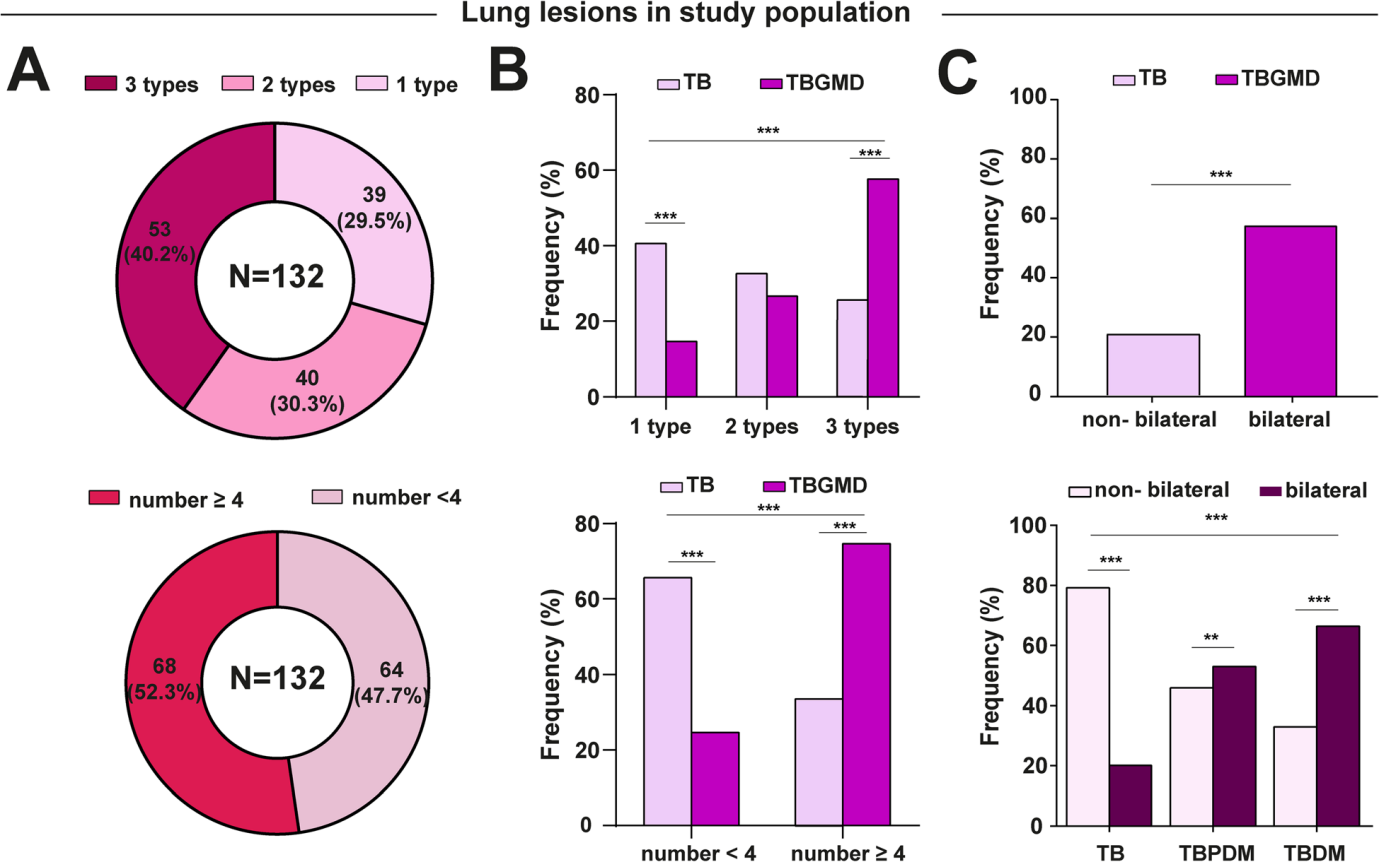
Distribution and frequency of type and number of lung lesions in tuberculosis patients with dysglycemia. **a** Stratification of patients with 1, 2 and 3 types of lung lesions and patients with the number of lung lesions ≥4. **b** Distribution of types of lung lesions (upper panel) and number of lung lesions ≥4 (lower panel) between the TB and TBGMD groups **(C)** Frequency of bilateral lesions in patients classified in TB and TBGMD (upper panel) or in those classified in TBPDM and TB-DM (lower panel). In **b** and **c**, data were compared between the groups using the Fisher’s exact test (2 × 2 comparisons) or the Pearson’s chi-square test (> 2 groups). TB: tuberculosis, GMD: glucose metabolism disorders, DM: diabetes mellitus PDM: prediabetes, FPG: Fasting plasma glucose, HbA1c: glycated hemoglobin, OGTT: oral glucose tolerance test. One type lesion: cavity (CV) or infiltrate (IN) or fibrous tract (FT); 2 lesion types: CV + IN or CV + FT or IN + FT; 3 lesion types: CV + IN + FT. Number of lung lesions ≥4: considering the total number of cavities + total number of infiltrates + total number of fibrous tracts. Only comparisons with significant *p*-values are displayed (*p < 0.05, **p < 0.01, ***p < 0.001)

Corroborating with the idea that dysglycemia is associated with worse radiographic presentation, the median values of FPG and HbA1c were significantly higher (*p* < 0.01) in TB patients affected with more lung lesion types (Fig. 2b). In addition, we found that 40.2% of TB patients had more than three lesions and 60% of those were simultaneously PDM or DM (Fig. 3a). Moreover, 52.3% of TB patients had more than 4 lesions; and > 70% of those also had hyperglycemia (Fig. 3a and b). TB patients with hyperglycemia more frequently exhibited bilateral lung lesions compared to those with normoglycemia (Fig. 3c).

**Fig. 4:**
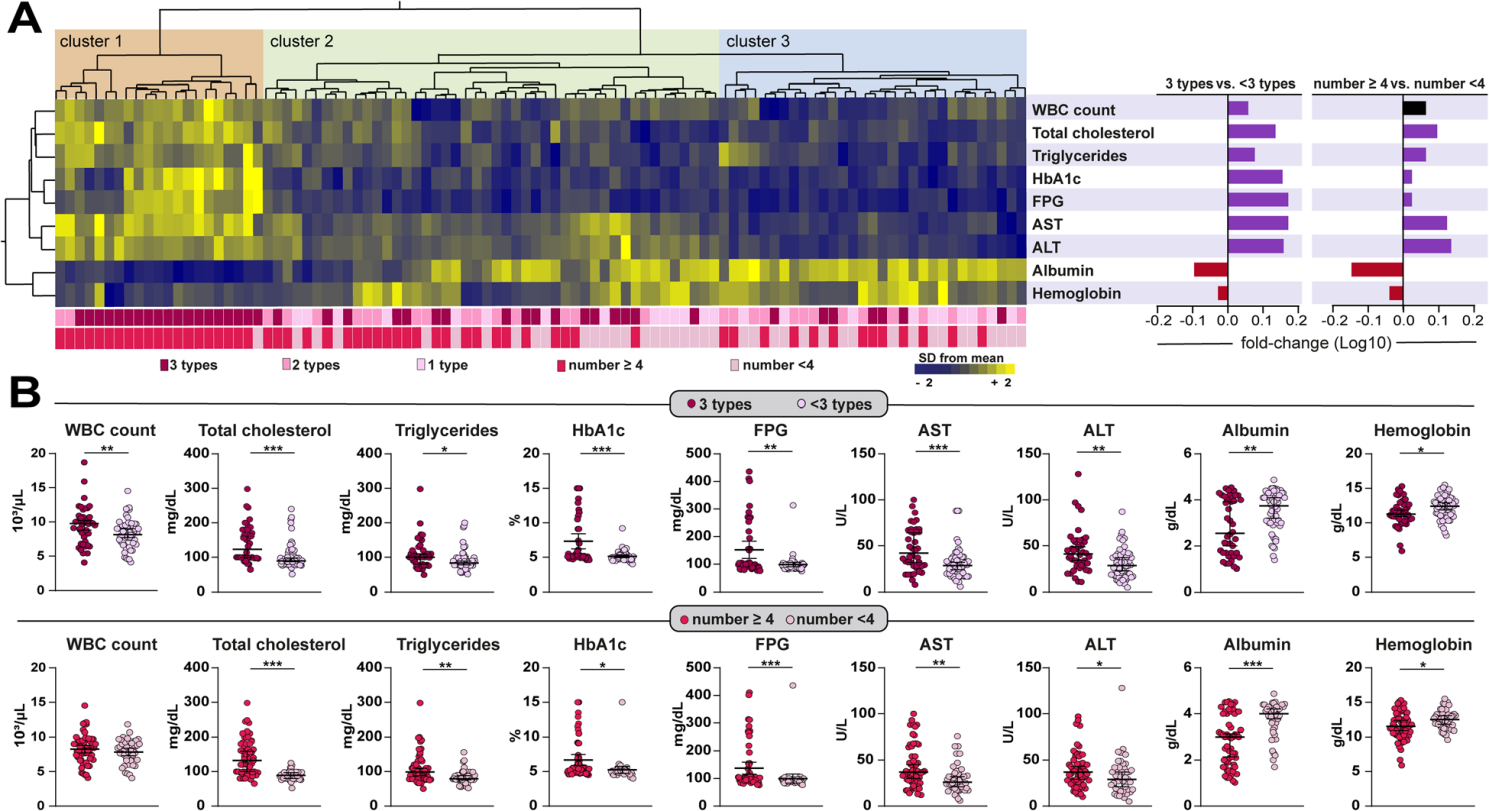
Biochemical profiles of pulmonary TB patients stratified by type and number of lung lesions. **a** Value of each parameter was Log10 transformed. Mean values for each indicated clinical group were z-score normalized and a hierarchical cluster analysis (Ward’s method with 100X bootstrap) was performed to illustrate the overall biochemical profiles. Fold differences (between the following groups: 3 types vs. < 3 types, and number ≥ 4 vs. number < 4) were calculated and statistically significant differences are highlighted in purple (positive) and red (negative). **b** Data represent median and interquartile ranges. The Mann-Whitney *U* test was employed to compare the values detected between the study subgroups. One type lesion: cavity (CV) or infiltrate (IN) or fibrous tract (FT); 2 lesion types: CV + IN or CV + FT or IN + FT; 3 lesion types: CV + IN + FT. Number of lung lesions ≥4: considering the total number of cavities + total number of infiltrates + total number of fibrous tracts. Only comparisons with significant p-values are displayed (*p < 0.05, **p < 0.01, ***p < 0.001).

Furthermore, we employed a hierarchical cluster analysis using a number of laboratory parameters measured in peripheral blood to test whether it was possible to identify a specific bio-signature that could characterize TB patients presenting with different types and numbers of radiographic lung lesions (Fig. 4a). This approach revealed that patients with 3 types of lung lesions and also with 4 or more lesions exhibited a distinct profile hallmarked by higher values of WBC counts, total cholesterol, triglycerides, liver transaminases as well as values of the DM screening tests (FPG, and HbA1c). The same subgroup of patients also exhibited lower levels of plasma albumin and hemoglobin compared to those from TB patients presenting with lower number and types of lesions (Fig. 4a). We next described in details the associations of biochemical and cellular parameters with number or types of lung lesions (Fig. 4b). We found that all parameters, except for the WBC counts in patients with> 4 lesions, were statistically different between the groups (Fig. 4b).

**Fig. 5:**
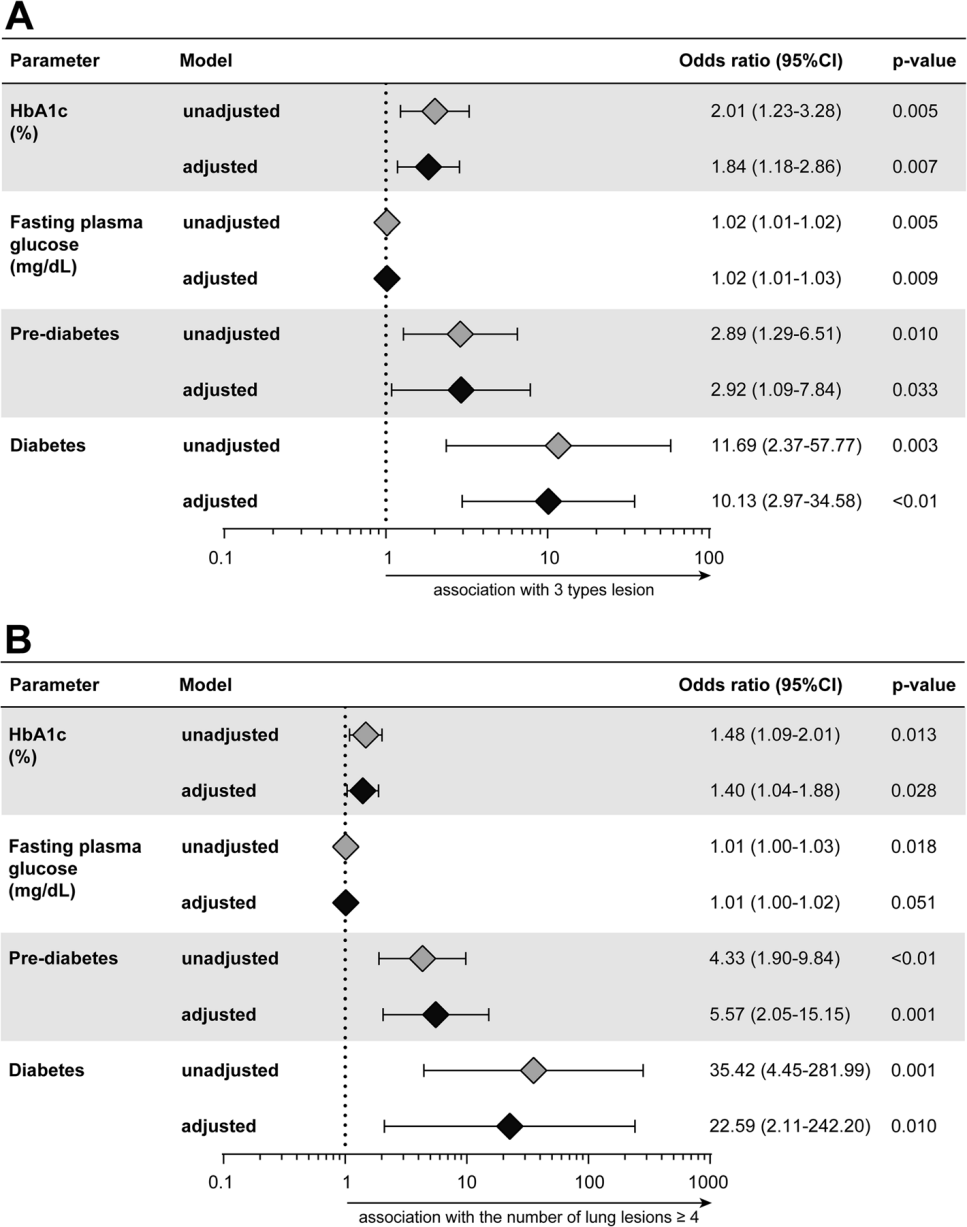
Multivariable regression analysis to evaluate association between HbA1c, fasting plasma glucose, prediabetes, and diabetes with diversity and number of lung lesions in pulmonary TB patients. Multivariable logistic regression analysis was conducted to test association between indicated variables and odds of presenting with 3 types of lung lesions (**a**) and the number of lung lesions ≥4 (**b**) in radiographic evaluation of TB patients. In univariate model, each variable was tested individually. The adjusted model included all the variables shown in the graphs in addition to age, sex, prior TB, ≥2+ AFB smear and hemoglobin level. HbA1c: glycated hemoglobin

Having demonstrated that hyperglycemia significantly affected the biochemical profile in peripheral blood, we further investigated whether presence of PDM or DM directly associated with the radiographic presentation of pulmonary TB using multivariable logistic regression analyses (Fig. 5). We first observed that increases of 1 unit in HbA1c and of FPG values reflected increased odds of having 3 pulmonary lesion types (Fig. 5a) or ≥ 4 lung lesions in TB patients (Fig. 5b), except for FPG which lost significance with the latter parameter after adjustment for age, sex, prior TB, hemoglobin levels and acid-fast bacilli ≥2+ in sputum smears. Secondly, presence of PDM or DM was independently associated with increased odds of presenting with these worse radiographic manifestations of pulmonary TB (Fig. 5a and b), after the statistical adjustments.

## Discussion

In this study, we demonstrated that hyperglycemia (DM and PDM) is strongly associated with worse radiographic manifestation of pulmonary TB in patients from Peru. The results showing association between DM and more increased severity of radiographic TB disease are similar to previously reported findings [13, 24], but the findings on PDM have not been published before. Herein, a large proportion of the patients who presented with three types of lesions (cavities, infiltrates and fibrous tracts), or with more than 4 lesions, also had hyperglycemia. It is possible that dysglycemic patients presented with more advanced TB disease, which could have impacted the radiographic characteristics as indicated by others [25]. Regional differences in epidemiology of both TB and hyperglycemia may result in distinct impact of the glycemic control status on the radiographic presentation of pulmonary TB, reported by studies performed in different countries.

We have previously reported that patients with hyperglycemia more frequently present with multiple TB-related symptoms compared to those with normoglycemia [11]. In addition, TB-DM patients more commonly required transfer from a primary care center to hospitals in order to have access to more complex care [11]. These reported results were obtained in different patient populations from Brazil. Of note, we failed to demonstrate an association between hyperglycemia and TB-clinical presentation in the present study performed in Peru. Again, discrepancies driven by epidemiologic, clinical and even microbiologic factors may explain the conflicting findings. Despite the differences in clinical presentation, several studies reported similar results to those reported here, showing increased mycobacterial loads in sputum smears in hyperglycemic vs. normoglycemic patients [11, 13, 26–29]. A multinational, harmonized, clinical study is necessary to formally demonstrate the effect of DM and/or PDM on TB disease presentation and to test if such effect is independent on the bacterial loads in sputum.

Our findings indicate that hyperglycemia is associated with significant increases in the number tuberculoid lesions and in the diversity of the lesion types, as well as with occurrence of bilateral lung disease, in agreement with previous studies [12, 14]. This increased degree radiographic extension of TB disease may result in a possible acceleration of pulmonary dysfunction [30]. Some authors have reported that cavitary lesions in hyperglycemic patients are generally unilateral, affecting only one side of the lung [15], which stands contrary to our findings. The reason for this discrepancy deserved further investigation. The mechanism of more severe radiological manifestations could be related to hyper-stimulation of leukocytes by both TB and the hyperglycemic state, resulting in a ‘premature aging’ of the lung [15] in patients with TB-DM comorbidity.

Our findings reveal that hyperglycemia was associated with a systemic pro-inflammatory state characterized by elevation of WBC counts and increased levels of liver transaminases, total cholesterol and triglycerides in serum, whereas albumin and hemoglobin levels were decreased, suggesting anemia. The hierarchical cluster analysis further demonstrated that the cellular and biochemical profile associated with hyperglycemia was also linked occurrence of increased number and diversity of lung lesions. We have recently described that TB-related anemia is associated with a distinct inflammatory profile that persists upon initiation of antitubercular therapy in a Brazilian cohort [23], but we have not previously tested the association between anemia and hyperglycemia in TB clinical or radiographic presentation. It is possible that progression of TB disease links these two apparently distinct pathophysiological conditions, hyperglycemia and anemia, through a mechanism that may involve chronic inflammation. Additional studies examining the intersection between TB, chronic inflammation, anemia and hyperglycemia are required to clarify this question.

Our study has some limitations. The analytical design did not allow us to investigate the temporary effect of TB infection on hyperglycemia, or vice-versa. Therefore, causal relationships cannot be established from this study. The cross-sectional design also prevented us to analyze the impact of TB treatment on the glycemic control or to answer whether diabetes therapy impacts TB treatment response. In addition, a different number of patients in each group of distinct glycemic status could have affected the accuracy of our findings in terms of confidence intervals. Nevertheless, the detailed analyses merging clinical, laboratorial and radiographic investigations demonstrated a significant association between glycemic status and the presentation of pulmonary lesions in TB patients. If validated by other studies, the results presented here reinforce the importance of performing glycemic control in TB patients.

## Conclusions

Hyperglycemia (both DM and PDM) was found to significantly increase the frequency and diversity of pulmonary lesions in patients with tuberculosis. Hyperglycemic TB patients were found to have significant alterations of the biochemical profile in peripheral blood, suggesting that there is a distinct metabolic state that likely contributes to the severity of pulmonary disease. These findings underscore the need for improved, accurate, screening and diagnosis of hyperglycemic conditions, and adequate glycemic control in patients with TB. Additional studies should be pursued to understand how the increased radiographic extension of TB observed in dysglycemic patients may affect pathogen transmission.

## Data Availability

The datasets used and/or analyzed during the current study available from the corresponding author on reasonable request.

## Abbreviations

ADA: American Diabetes Association
AFB: Acid-Fast Bacilli
ATT: Antitubercular treatment
BCG: Bacillus Calmette–Guérin
BMI: Body Mass Index
DM: Diabetes mellitus
FPG: Fasting plasma glucose
Hb: Hemoglobin
HbA1c: Glycated Hemoglobin
HHC: Household contact
IQR: Interquartile range
L-J: Löwenstein-Jensen
MDR: Multi Drug Resistant
Mtb: *Mycobacterium tuberculosis*
NTP: National TB Program
OGTT: Oral glucose tolerance test
PDM: Prediabetes
SEIS: Socios En Salud Informatic System
TB: Tuberculosis
TG: Triglycerides
WHO: World Health Organization
